# Supplementary material for: Assessing the chemical profile and biological potentials of Tamarix smyrnensis flower extracts using different solvents by in vitro, in silico, and network methodologies
Source: PLoS One. 2025 Dec 1;20(12):e0337420. doi: 10.1371/journal.pone.0337420 (PMC12668501; doi:10.1371/journal.pone.0337420)
Supplement: S1 Table — (DOCX) [file pone.0337420.s001.docx]

Table S1. Antioxidant activity results of plant extracts (n = 3).

| Parameter | Solvent | Replicate 1 | Replicate 2 | Replicate 3 |
| --- | --- | --- | --- | --- |
| DPPH (µg/mL) | Methanol | 26.5 | 28.9 | 28.0 |
|  | Ethanol | 24.3 | 26.9 | 24.3 |
|  | Ethyl Acetate | 60.5 | 68.7 | 68.4 |
| Metal Chelating (mg/mL) | Methanol | 51.2 | 47.3 | 51.0 |
|  | Ethanol | 78.2 | 82.9 | 79.4 |
|  | Ethyl Acetate | 171.1 | 176.2 | 175.0 |
| CUPRAC (µg/mL) | Methanol | 36.9 | 34.3 | 36.7 |
|  | Ethanol | 56.1 | 59.3 | 59.4 |
|  | Ethyl Acetate | 149.2 | 153 | 152.2 |
