# Supplementary material for: Assessing the chemical profile and biological potentials of Tamarix smyrnensis flower extracts using different solvents by in vitro, in silico, and network methodologies
Source: PLoS One. 2025 Dec 1;20(12):e0337420. doi: 10.1371/journal.pone.0337420 (PMC12668501; doi:10.1371/journal.pone.0337420)
Supplement: S3 Table — (DOCX) [file pone.0337420.s003.docx]

**Table S3**. Docking validation summary showing RMSD values of ligands re-docked into their respective active sites, confirming protocol reliability.

| Target PDB | Validation Type | Reference (Liganded) PDB | Ligand | RMSD (Å) |
| --- | --- | --- | --- | --- |
| 5IKQ | Native re-docking | 5IKQ | Native ligand | 0.38 |
| 1N45 | Cross-redocking | 3CZY | Cognate ligand | 1.29 |
| 1V04 | Cross-redocking | 3SRG | Cognate ligand | 0.80 |
